# Symmetry Splitting of Equivalent Sites in Oxide Crystals and Related Mechanical Effects

**DOI:** 10.6028/jres.067A.029

**Published:** 1963-08-01

**Authors:** J. B. Wachtman, H. S. Peiser, E. P. Levine

## Abstract

Changes in the symmetry of a crystal caused by an applied strain have been used to show in what circumstances an internal friction peak can result from the motion of isolated point defects. General rules are given to make the prediction, and these are applied to several structures of common oxides. The prediction for rutile is compared with experimental results which are interpreted by the movement of titanium ions between interstitial positions in the structure.

## 1. Introduction

Point defects, such as vacancies and interstitial atoms, are important in the deformation of crystalline solids. They cause Nabarro-Herring creep and interact with dislocations. Knowledge on jump frequencies between neighboring defect sites is therefore interesting and may be obtainable from internal-friction studies. From the disappearance of the internal-friction peak in *α*-iron when deprived of carbon and nitrogen, Snoek [1]^1^ proposed his theory on the stress-induced jumping of interstitial atoms in body-centered cubic structures. As summarized by Berry [2], this model has been diversely applied to the study of other *bcc* metals such as tantalum containing carbon, oxygen, or nitrogen, and vanadium containing oxygen or nitrogen. Many new problems arise. It is natural, for example, to ask if such studies can be useful in ceramic oxides and other nonmetallic crystals.

The purpose of this paper is to present rules to show in what circumstances isolated point defects can contribute to internal friction, and to predict for specific oxides the types of defect which can be expected to give an internal-friction peak. There exist similar phenomena which by this stated purpose are excluded from discussion. For example, a point defect, such as an oxygen vacancy, may be constrained to remain in the neighborhood of a relatively immobile dislocation or defect such as a substitutional impurity atom. The motion of this oxygen vacancy can be studied by internal friction. The activation energy determined in such an experiment may differ from that for a free vacancy. Some discussion of pairs of point defects including pairs of atoms making up a split-interstitial site can be found elsewhere [2,3].

The approach in this paper might be termed the crystallographic method and should be compared with the “elastic dipole” being used by Nowick and co-workers [4] by analogy with the electric dipole because of the insight obtained by comparing dielectric and mechanical relaxation. The local distortion which interacts with a homogeneous strain does not have the symmetry of a true dipole, and “elastic dipole” should be understood as denoting a centrosymmetric local distortion and not as a type of dipole.

The “elastic dipole” method has developed out of work on mechanical and related electrical effects by A. S. Nowick and collaborators including B. S. Berry, R. W. Dreyfus, W. R. Heller, and R. B. Laibowitz [3,4,5,6,7,8]. A general paper is being prepared by Nowick and Heller [9].

The essential ideas of the crystallographic method have been previously introduced [10]. The present paper gives a fuller discussion and presents a series of applications.

## 2. Theory

### 2.1. Equivalence of Sites Under Arbitrary Strain

**Figure 1:**
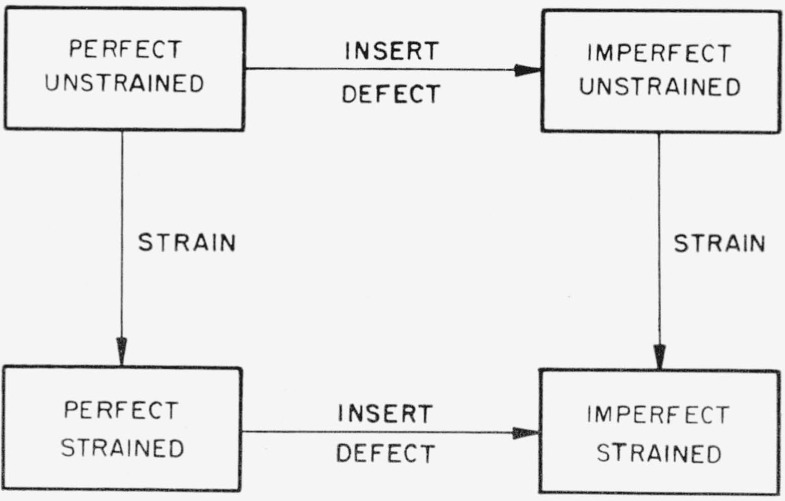
Scheme showing two hypothetical ways of producing an imperfect, strained crystal from a perfect, unstrained crystal.

The box in the upper left-hand corner of figure 1 represents a perfect, strain-free crystal. We are concerned with the state of affairs in a crystal containing defects which is placed under an externally imposed homogeneous strain. The approach used in the elastic-dipole method, as given, for example, by B. S. Berry [2], although he does not use the term “elastic dipole”, is to begin with the perfect, unstrained crystal, next to consider a defect introduced, setting up a local distortion, and finally to consider how the distorted portion of the crystal interacts with an externally applied strain. In the crystallographic method, the perfect crystal is considered to be homogeneously strained and a point defect is then inserted in either of two positions which were equivalent in the unstrained, perfect crystal. If the positions in the strained, perfect crystal were still equivalent, no internal friction would result, because a point defect would have the same energy if placed on either position and should therefore have no preference for either position. If, however, these positions in the strained, perfect crystal are inequivalent, the point defect should have different energies when placed on the two positions and a preferred distribution should result, leading to internal friction if the jump rate of the point defect is approximately equal to the frequency of the alternating strain. Therefore, only the behavior of the perfect crystal under strain need be considered in predicting the possible existence or absence of an internal friction peak which is caused by a point defect which can occupy a given set of equivalent positions in the crystal. When a peak is permitted on symmetry grounds, its magnitude may or may not be adequate for detection by a given experiment.

Now consider the effect of a homogeneous strain on a single crystal. We can think of a crystal as made up of a set of identical unit cells. Strain distorts these cells but, if the strain is homogeneous, all the cells will distort in the same way and so remain equivalent. Therefore, we need only consider what happens in one unit cell. It is convenient to choose the smallest possible cell—the primitive cell—which can be repeated in space to generate the crystal. In so doing, we have exploited the translation symmetry of homogeneous strain. There remains one other general symmetry property of homogeneous strain; it is always centrosymmetric. Thus a center of symmetry in a crystal cannot be removed by homogeneous strain and two positions in a crystal which are equivalent due to a center of symmetry cannot be made inequivalent by homogeneous strain.

These facts form the basis of *Rule 1:* Internal friction cannot occur if there is only one position per primitive cell (i.e., per lattice point in the strict crystallographic sense) or if there are only two, which are related by a center of symmetry. Internal friction should occur for some state of strain if there are two positions unrelated by a center of symmetry or if there are three or more positions.

**Figure 2:**
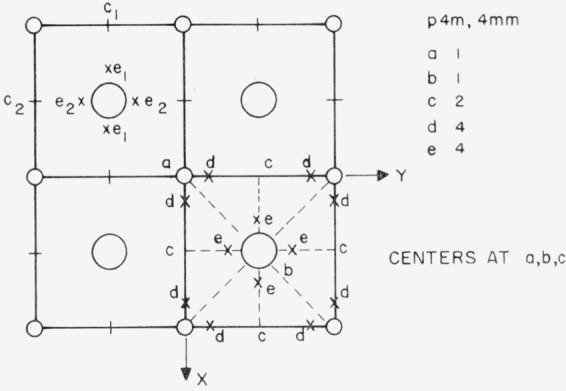
A two-dimensional illustration of a crystal showing four unit cells. Each type of position is given the letter symbol as in ref. 11 and the number per unit cell is given.

To illustrate the application of this rule, consider the hypothetical two-dimensional oxide, MO, shown in figure 2. The positions are labeled according to the conventions used in the International Tables for X-ray Crystallography [11]. The metal ions are at positions *a* at the corners of the cells. There is only one *a*-type position per cell. Thus, all metal ion sites must remain equivalent under homogeneous strain. The oxygen sites also remain equivalent under homogeneous strain because there is only one *b*-type site per lattice point. Thus, we could not expect internal friction for either type of vacancy or for a substitutional impurity. The situation is different for interstitial ions, however. The largest interstitial position is labeled *c* and there are two per cell, *c*_1_ and *c*_2_. There is no center of symmetry to relate them, so some state of strain must exist which will split the set of *c*-type sites into two inequivalent subsets of equal size. In this case, we speak of a splitting factor of two; in the case of no splitting, we speak of a splitting factor of one. Now consider the four *e*-type positions. Interstitial hydrogen might take up such a position, close to the oxygen and equidistant from the two metal ions. The center of symmetry at the oxygen position keeps *e*_1_ equivalent to *e*_1_, and *e*_2_ equivalent to *e*_2_ after strain. Here we have a set of four sites per primitive cell splitting into two sets of two each and again we have a splitting factor of two.

The type of position actually occupied by a point defect must, of course, be used in these considerations and this is not always obvious. An impurity atom at a site (say *a* of fig. 2) may shift and form covalent bonds with some but not all of its initially equivalent neighbors, in which case it would have the symmetry of one of the neighboring special positions of lower symmetry (say *d* of fig. 2). Even when covalent bonds are not formed, the same result may be caused by the Jahn-Teller effect. In either case, alternating strain could then cause jumps from one of the *d*-type positions neighboring an *a*-type position to another. A more complicated situation would arise if the *a*-type position also split into subsets under a suitable strain (this is not true for our hypothetical oxide, MO, but a more complicated example could be given for which it is true.) One would then have to consider jumps, characterized by activation energy *E_d_*, between *d*-type positions neighboring an *a*-type positions and jumps, characterized by an activation energy *E_a_*, between *a*-type positions. If *d*-type positions are occupied because of the formation of covalent bonds, *E_d_* and *E_a_* will both probably be large and there is no general reason to assume one is much larger than the other. For Jahn-Teller symmetry lowering, however, it appears that *E_d_* would usually be appreciably less than *E_a_.* Jahn-Teller effects are known to exist [12] and sites of lower symmetry for “substitutional” atoms may be preferred by as much as about 0.1 ev. This is a rather low value compared to the activation energy for motion of point defects of the type important in material transport processes, which is found [13] to range upward from about 0.4 ev for the alkali halides and is probably higher in the refractory oxides. If *E_d_* is only a small fraction of *E_a_*, one might expect to see two internal friction peaks in a curve of internal friction as a function of temperature at constant frequency, *f*. One peak should occur at a temperature for which the jump frequency, *v_d_*, of the impurity atom between *d*-type positions neighboring a single *a*-type position approximately matches the experimental frequency. At this temperature, the jump frequency, *v_a_*, between *a*-type positions would be negligibly small if *E_a_* is several times larger than *E_d_.* If the temperature is raised enough to make *v_a_* approximately equal *f*, the value of *v_d_* will become much larger. In effect, the motion over *d*-type sites averages out, so far as the internal friction peak caused by jumps between *a*-type sites is concerned, and for the discussion of symmetry restrictions on the high temperature peak, it is possible to treat the impurity atom as if it occupied the *a*-type sites rather than the *d*-type. This argument underlies our subsequent discussion of the symmetry conditions for internal friction associated with various point defects in the refractory oxides. For example, we discuss oxygen vacancies as if they occupied sites with the full symmetry of the oxygen position whereas it seems quite possible that Jahn-Teller symmetry lowering occurs. Throughout the discussion we assume that jumps between the sites of full symmetry have an appreciably higher activation energy than jumps between neighboring sites of lower symmetry and we limit our consideration to the high temperature range for which the jumps between sites of full symmetry could give internal friction peaks if permitted by symmetry.

### 2.2. Equivalence of Sites Under Specific States of Strain

The splitting factor is simply the number of inequivalent subsets into which the original set of initially equivalent positions splits. Usually the splitting will be into equal subsets (i.e., into subsets of equal size.) Thus, a set of six positions would be expected to split into two subsets of three positions or into three subsets of two positions or into six subsets of a single position. Under very special conditions, the set might split into a subset of two and a subset of four positions. Such splittings into unequal subsets do occasionally occur. We proceed to formulate a rule for the case of splitting into equal subsets. If this rule is blindly applied to a case of splitting into unequal subsets, it will sometimes signal trouble by giving nonintegral values of the splitting factor. These situations can also be treated by the present methods, but we omit detailed discussions to save space.

**Figure 3:**
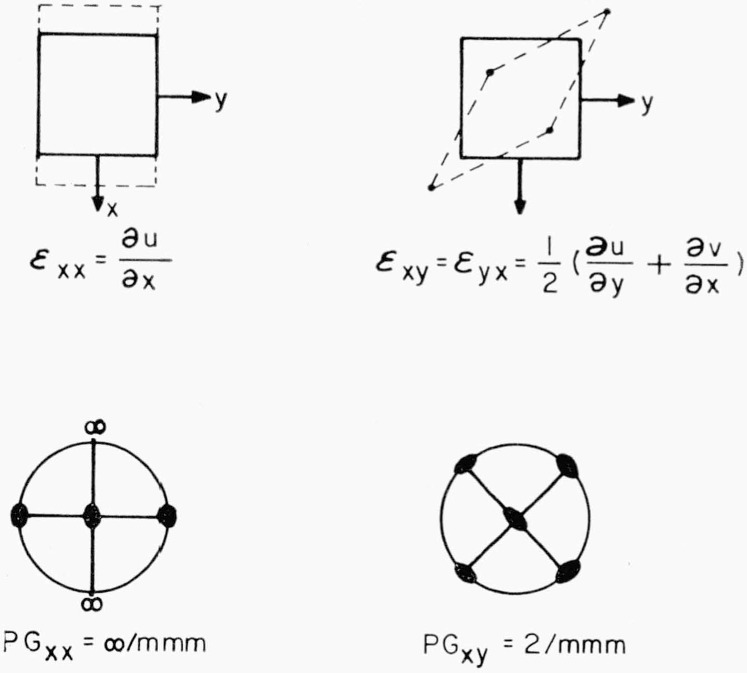
The effect of tensile strain, *ϵ_xx_*, and shear strain, *ϵ_xy_*, on a square. Stereograms show the point group associated with each strain in three dimensions.

For the case of the splitting into equal subsets, we have *Rule 2:* The splitting factor of equivalent positions is equal to a fraction whose numerator is equal to the ratio of the order of the point group of the unstrained crystal to the order of the position point symmetry in the unstrained crystal; the denominator is the same ratio evaluated for the strained crystal. The quantity in Rule 2 is just the fraction whose numerator is the number of equivalent positions in a set in the unstrained crystal and whose denominator is the number of positions in a subset in the strained crystal. The expression in the numerator requires a little explanation. If an atom is put down in the unit cell of a crystal at a point having no position symmetry (i.e., a general position) and then acted upon by the symmetry elements of the crystal, a set of *n* atoms will be generated, which is equal to the order of the point group of the crystal, written *n*(PG). If the atom were put down at a position having some symmetry (special position), called the position point symmetry, then some of the positions generated by the action of the symmetry operations would coincide. To take this into account, we must divide out the number of coincident positions to obtain the number of distinct positions. Thus, the numerator is the number of equivalent positions in the general case divided by the number which coincide when the position has position point symmetry denoted by PPS. The denominator is the same quantity evaluated in the strained crystal. Subscript zero will be used to refer to the unstrained crystal and subscript one to refer to the strained crystal. We shall give an application of Rule 2, but first let us consider how to evaluate the action of a specific strain on PG_0_ to obtain PG_1_, and on PPS_0_ to obtain PPS_1_. Consider the symmetry of the components of homogeneous tensile strain of Shear strain; i.e., what is the highest point symmetry (not necessarily crystallographic symmetry) Consistent with the existence of a given strain. Take a point at the center of a sphere. Initially this point will have a symmetry which includes any possible crystallographic point symmetry; we wish to know the maximum symmetry possible after each component of strain has been applied. The two types of strain components are illustrated in two dimensions (for ease of visualization) in the upper part of figure 3, which shows the distortion of a square under tensile strain and under shear strain. The lower part of figure 3 shows the three dimensional point group (convention as in reference 11) which a sphere would possess after each of these strains. The point group for tensile strain, PG_xx_= ∞/mmm, contains an infinite fold axis. That is, a rotational symmetry axis of any order is consistent with the axis of a tensile strain although axes higher than 6-fold cannot exist in crystals. The point group for shear strain, PG_xy_=2/mmm (abbreviated mmm), is seen to differ from PG_xx_ in containing no axis higher than 2-fold and in the orientation of the symmetry elements which it does contain. Any state of strain can be made up of combinations of tension and shear strains. The symmetry elements in a strained crystal will be those common to the unstrained crystal and to the strain.

Curie’s principle [14] postulates that a homogeneously strained crystal will have the symmetry elements common to the point group of the unstrained crystal and to the strain for the given mutual orientation of the symmetry elements of the initial crystal point group and of the imposed strain. Curie’s principle has been discussed especially by Shubnikoy [15] and Koptsik [16]. We assume that this principle applies to position point symmetry, i.e., that the point symmetry of a position in the strained crystal consists of the symmetry elements common to the original position point symmetry and to the strain (again in the given mutual orientation).

**Figure 4:**
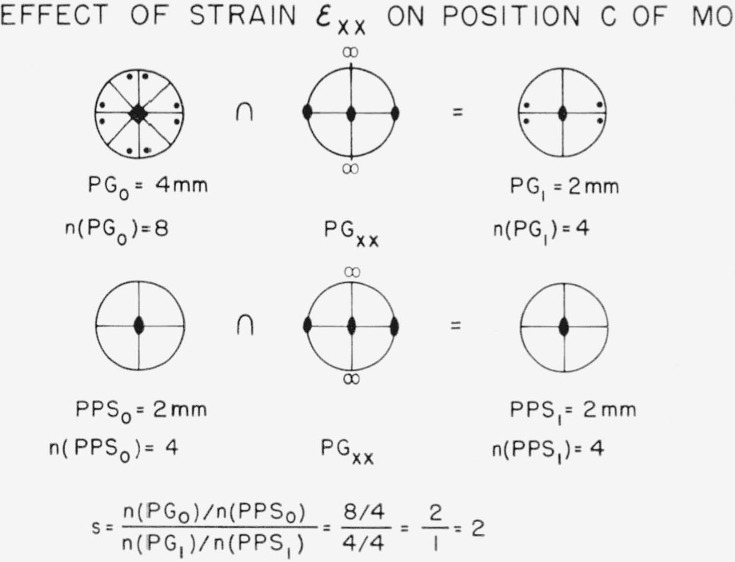
An illustration of the effect of tensile train in lowering the point group symmetry and position point symmetry. The point group of the tensile strain, PG_xx_, is drawn for three dimensions. Its application to two dimensions is simple; only the subgroup of operations appropriate to the plane under discussion need be considered.

**Figure 5:**
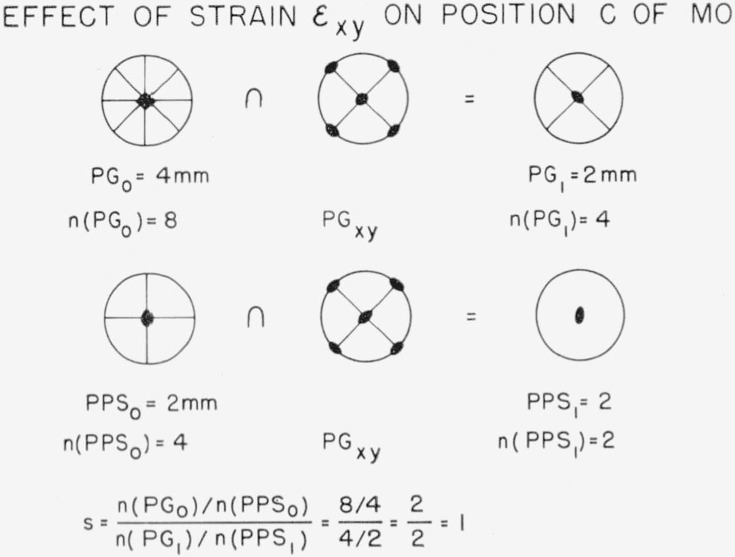
An illustration of the effect of shear strain in lowering the point group symmetry and position point symmetry.

Let us apply these ideas to the specific case of position *c*, the largest interstitial hole, in our hypothetical oxide, MO. Figure 4 shows the effect of strain *ϵ*_xx_. The point group of the unstrained crystal, PG_0_, is 4mm, which means a 4-fold axis intersected by two sets of mirror planes. This is shown in a pattern in which the small solid square represents the 4-fold axis and the heavy lines represent the mirror planes. The points show a set of eight equivalent general positions generated from one position so the order of the group is 8. We now take the symmetry elements in common with the point group of the strain *ϵ*_xx_, that is, we take the intersection, denoted by the symbol ∩, of the two groups of symmetry operations. There is a logical difficulty (but no practical difficulty) in taking the symmetry elements common to a three dimensional point group, PG_xx_, and a two dimensional point group, 4mm. One simply takes those elements of PG_xx_ which have meaning with respect to the plane of 4mm and finds which of these are also present in 4mm; the result is the intersection desired. The common elements are one 2-fold axis and two mirror planes giving us 2mm (equivalent to mm2) for PG_1_ with order 4. The position *c* has two mirror planes which intersect in a 2-fold axis so that PPS_1_ is 2mm with order 4. Taking the intersection with PG_xx_, the point group of *ϵ*_xx_, simply gives us PPS_1_ = PPS_0_. The splitting factor is thus 2. Now consider the action of the shear strain *ϵ*_xy_, shown in figure 5 (the reference axes for components are Cartesian). The two figures on the left of the diagram are the same as in figure 4, but note the change in the point group of the strain. Taking the intersection of PG_0_ with the point group PG_xy_ again gives us 2mm for PG_1_. The intersection of PG_xy_ with PPS_0_, however, contains only the 2-fold axis so that *n*(PPS_1_)=2. The splitting factor thus equals 1. In other words, internal friction would not be allowed for *ϵ*_xy_, but it would be permitted for *ϵ*_xx_.

This rapid sketch indicates how the real, three-dimensional oxides can be treated.

### 2.3. Calculation of Possible Subgroups

**Figure 6:**
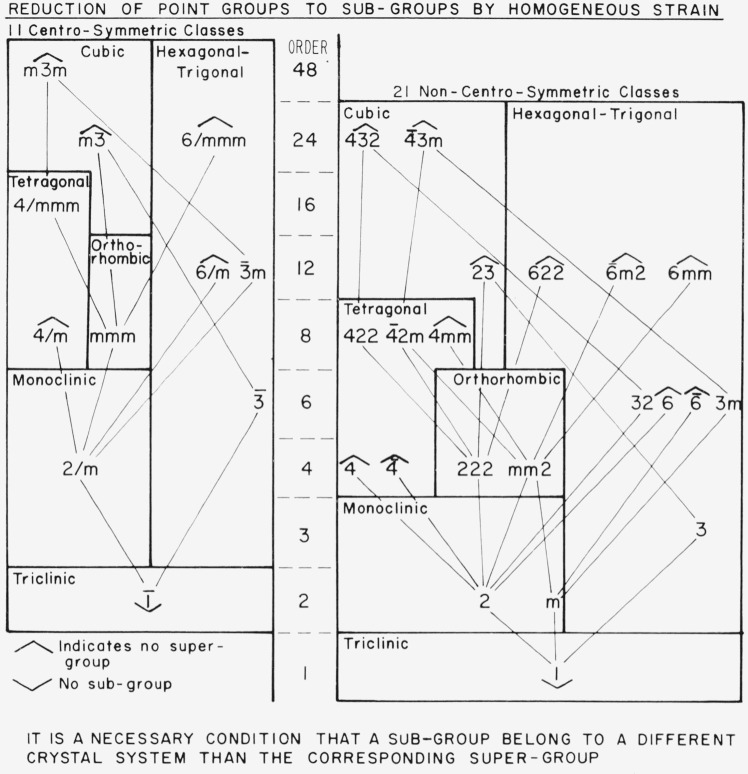
Reduction of point groups to subgroups by homogeneous strain.

There is a general table [10] which can be used to speed up the calculations somewhat. This table is shown in figure 6. There are only 32 crystallographic point-groups and homogeneous strain can only lower a given point group to one of its subgroups. Also, strain cannot change the crystal property of being centrosymmetric or non-centrosymmetric, so that the table splits into two separate parts. Further conditions arise because no strain can eliminate a two-fold axis perpendicular to or a plane of symmetry parallel to a three-, four-, or six-fold axis without also destroying the higher axial symmetry. Reduction to a subgroup turns out always to be accompanied by a change of crystal system (and a change in Bravais Lattice). This statement follows from the fact that even the most general strain in a crystal can be represented by a triaxial ellipsoid referred to Cartesian axes. A three-fold or higher axis restricts the triaxial ellipsoid to an ellipsoid of revolution which has mirror planes in all planes parallel to the unique axis and two-fold axes in all directions perpendicular to the unique axis. When all these conditions are taken into account, the table shown results. Point group 4/mmm, for example, can go only to mmm in the orthorhombic system or further to 2/m in the monoclinic system or further to 1 in the triclinic system. Point group 23 can either degrade to 3 or 222, but the former can only go to 1, whereas 222 can retain one of its diads independently of the other two.

## 3. Predictions for Specific Structures

### 3.1. Corundum-Type Structures (A1_2_O_3_)

**Figure 7:**
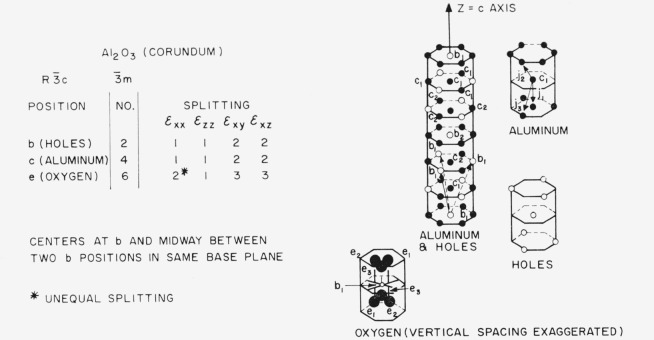
Splitting factors for selected positions in the corundum structure.

Let us turn to some specific refractory oxides and other structures of interest, beginning with aluminum oxide in the stable, alpha form. This has the corundum structure, space group $R\bar{3}c$, point group $\bar{3}m$, with 3 A1_2_O_3_ in the rhombohedral unit cell. The calculations for this structure follow the lines of the example just given; the results are shown in figure 7 with a drawing of the aluminum and hole positions. The drawing has been slightly idealized, following Kronberg [17], by showing the aluminum ions in planes instead of being slightly displaced; the results, however, hold for the real structure. Starting at the hole in the base of the cell marked *b*_1_ and moving along the *c* axis, we come to two aluminum ions, a hole, two more aluminum ions, and then the hole in the base of the next cell. The other aluminum ions and holes are arranged in identical chains which are parallel, but which have been moved up or down one position spacing. The oxygen ions are on hexagonal close-packed sheets at right angles to the aluminum chains. Our table shows that the tensile strains *ϵ*_xx_ or *ϵ*_zz_ cause no splitting of the sets of hole positions or aluminum positions. Shear strain causes a splitting into two equal subsets. The holes are labeled *b*_1_ and *b*_2_. A*b*_1_ position is shown with its 6 neighboring *b*_2_ positions. One might expect an inert gas such as helium, to occupy the holes and to diffuse by *b*_1_ to *b*_2_ type jumps. Internal friction experiments using shear strain might thus provide information of direct interest concerning the diffusion of noble gases in corundum. The set of aluminum positions splits into the subsets *c*_1_ and *c*_2_. A*c*_1_ position is shown with the neighboring *c*_2_ positions in the two adjacent sheets (all aluminum positions in the same sheet are *c*_1_ positions). There are three distinct types of jumps from a *c*_1_ position to a *c*_2_ position: *j*_1_ is parallel to the *c* axis and passes between three equidistant oxygen ions; *j*_2_ and *j*_3_ must pass around an oxygen ion. In an internal friction experiment, we would have a relaxation process taking place by three parallel paths and the fastest path would be rate-determining. In diffusion, a series of steps is involved and the slowest step in the particular series would be rate determining. The usefulness of internal friction relative to cation diffusion in corundum is thus problematical and further work is needed on the combination of jumps required in diffusion. An interesting experiment involving aluminum positions would be the attempt to observe the interchange of aluminum and chromium ions in ruby by doing an internal friction experiment in shear. Turning now to the set of oxygen positions, we see that this splits into three subsets, *e*_1_, *e*_2_, and *e*_3_. Here again a detailed theory of the combination of jumps involved in diffusion is needed, but it seems that some information of interest could be gained. In summary, for corundum it appears that internal friction experiments should be of direct interest to noble gas diffusion and very probably of interest in relation to oxygen diffusion (if nonstoichiometric alumina exists as various workers have suggested), but that the relation of internal friction to cation diffusion is problematical.

### 3.2. Periclase-Type Structures (MgO)

**Figure 8:**
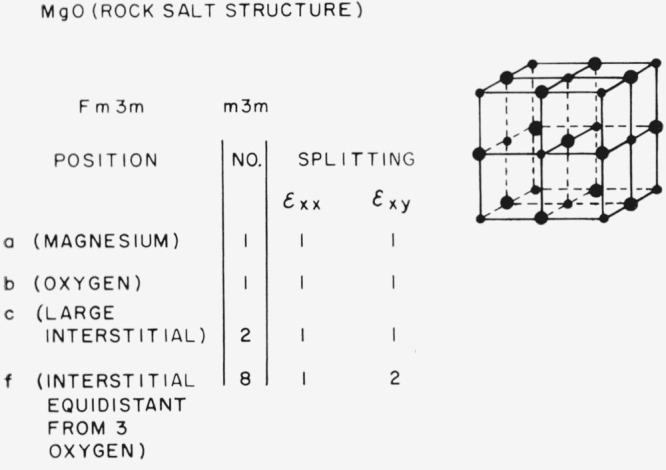
Splitting factors for selected positions in the rocksalt structure.

The results for MgO are shown in figure 8. There are just one magnesium ion and one oxygen ion per primitive cell so that no splitting occurs for either position. There are two large interstitial positions per primitive cell, but they are related by a center of symmetry so that no splitting takes place. The set of interstitial positions equidistant from three oxygen atoms does split into two subsets, but it seems unlikely that any impurity would occupy these positions. It thus appears that internal friction experiments are unlikely to give any information about isolated point defects in MgO. Pairs of point defects, however, are subject to different symmetry conditions as previously mentioned and internal friction experiments should be quite useful in the study of such pairs as a trivalent ion associated with a magnesium vacancy. There is also the previously mentioned possibility of the lowering of symmetry by the Jahn-Teller effect and the accompanying possibility of mechanical relaxation.

### 3.3. Rutile-Type Structures (TiO_2_)

**Figure 9:**
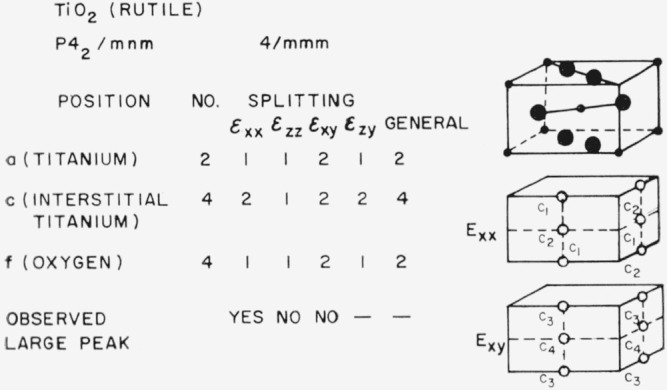
Splitting factors for selected positions in the rutile structure.

The situation for rutile is shown in figure 9. Here splitting exists for the titanium positions, the oxygen positions and the positions which interstitial titanium would be expected to occupy. The patterns of splitting are different and internal friction experiments seem to offer a good way to get information about the type of defect present in lightly reduced rutile. Data have been reported from two investigations [18, 19] which are in good agreement with each other. A peak was found in vacuum-reduced rutile which was not present when the specimen was stoichiometric. The peak occurs under strain *ϵ*_xx_ so that isolated oxygen vacancies are not responsible because the splitting factor for oxygen positions under strain *ϵ*_xx_ is one. The splitting factor for titanium interstitial positions under *ϵ*_xx_ is two and the existence of the peak suggests that the predominant type of point defect in vacuum-reduced rutile is the titanium interstitial. No peak is observed under strain *ϵ*_xy_, although the splitting factor for titanium interstitial sites is two. The interpretation of the defect causing the observed peak is thus uncertain; the interpretation which appears most probable is that the defect responsible is either a titanium interstitial (with a contribution to the internal friction under *ϵ*_xy_ too small to be observed) or a pair of titanium interstitials. Such a pair, with one interstitial in the edge of a (100) face and the other in the center of an adjacent (010) face would [18] give a peak under *ϵ*_xx_, but not under *ϵ*_xy_ or *ϵ*_zz_.

### 3.4. Fluorite-Type Structures (ThO_2_)

**Figure 10:**
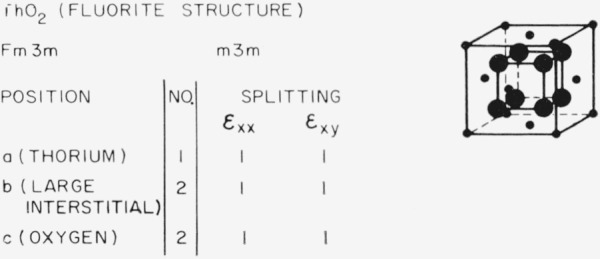
Splitting factors for selected positions in the fluorite structure.

Turning now to thoria with the fluorite structure shown in figure 10, we see that the thorium positions, the oxygen positions, and the largest interstitial positions do not split under any strain so that this structure is not a promising one for internal friction studies of isolated point defects unless Jahn-Teller symmetry lowering leads to symmetry permitted internal friction as previously discussed. An internal friction peak attributed to pairs composed of a substitutional calcium ion and an oxygen vacancy has been reported [20].

### 3.5. Diamond-Type Structures (C)

**Figure 11:**
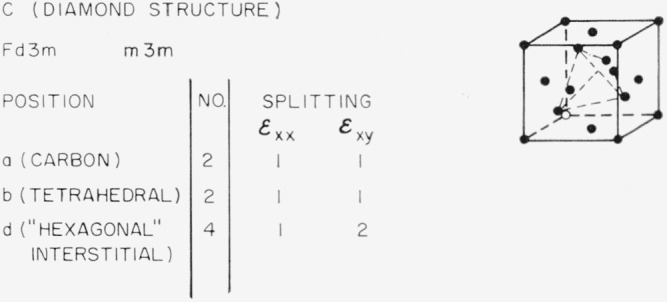
Splitting factors for selected positions in the diamond structure.

The next structure to be considered is the diamond structure shown in figure 11. The carbon positions do not split, nor do the larger holes called the tetrahedral interstitial sites. There are, however, smaller interstitial sites which a small ion might occupy. Weiser [21] has calculated the binding energy for several interstitial ions in either the tetrahedral or the “hexagonal” interstitial position in germanium or silicon which have the diamond structure. He concludes that for lithium, the “hexagonal” site is more probable than the tetrahedral site. This gives the opportunity for an interesting internal friction experiment, as the lithium ions should not contribute to internal friction if they are in tetrahedral sites, but are permitted by symmetry to do so if they are in “hexagonal” sites.

Southgate [22] has found an internal friction peak associated with oxygen in silicon. This could occur if the oxygen occupies “hexagonal” interstitial positions, but another possibility is that the oxygen is present in the tetrahedral holes, but occupies a special position of lower symmetry than the tetrahedral position. This is analogous to the substitutional positions *a* and *d* of our hypothetical two-dimensional oxide, MO, discussed previously. Infrared spectra indicate [23, 24] that the oxygen atom is in the tetrahedral hole, but is more tightly bound by covalent bonds to two of the four tetrahedrally situated silicon atoms; the oxygen atom thus occupies a position of lower symmetry and the occurrence of an internal friction peak is then consistent with the present theory. One can look upon the existence of an internal friction peak caused by isolated oxygen interstitials in silicon as proof that they do not occupy sites with the full symmetry of the tetrahedral interstitial position.

### 3.6. Perovskite-Type Structures (BaTiO_3_)

**Figure 12:**
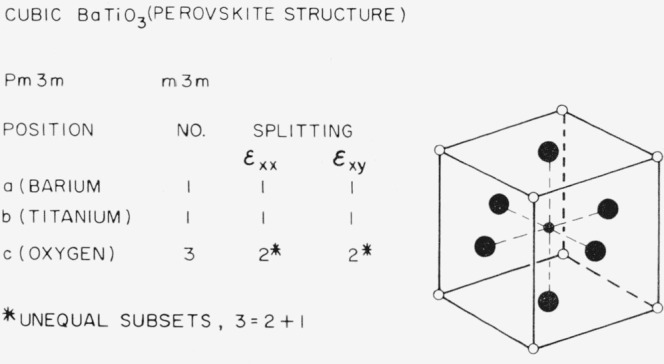
Splitting factors for selected positions in the perovskite structure.

The perovskite structure is shown in figure 12. The oxygen positions split and this might be an interesting structure to study.

### 3.7. Wurtzite-Type Structures (BeO)

**Figure 13:**
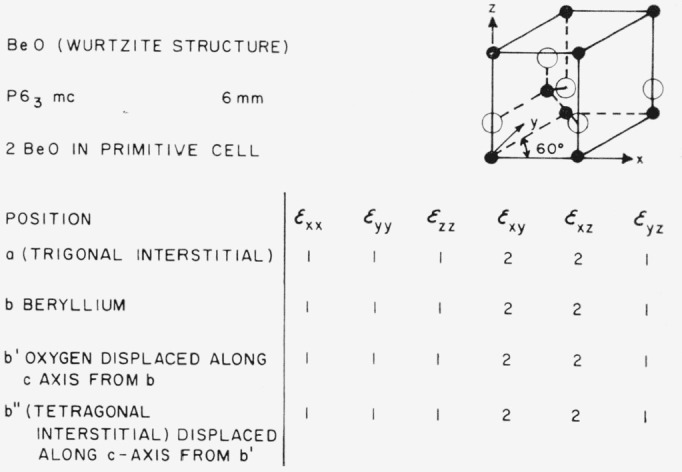
Splitting factors for selected positions in the wurtzite structure.

The wurtzite structure, which is the structure of both BeO and ZnO is shown in figure 13. Two of the components of shear strain cause splitting of anion, cation, and interstitial positions. This structure thus appears quite suitable for internal friction studies using the appropriate shear strain.

### 3.8. Spinel-Type Structures (MgAl_2_O_4_)

**Figure 14:**
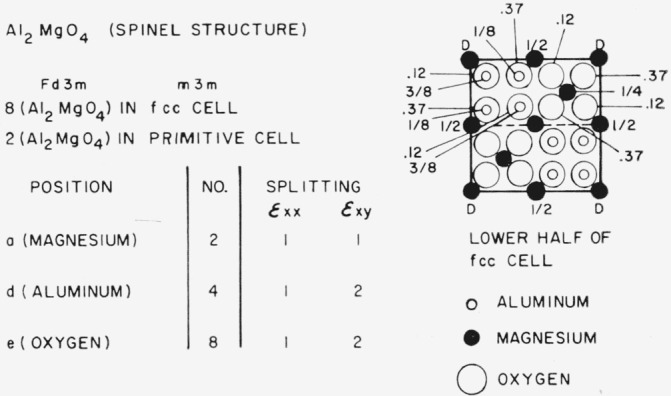
Splitting factors for selected positions in the spinel structure.

Finally, the spinel structure is shown in figure 14. The magnesium positions do not split, but the aluminum and the oxygen positions do. Spinel is known to form a solid solution with A1_2_O_3_ and internal friction studies might be of interest.

## 4. Summary

In summary, internal friction appears to offer a unique method of obtaining information on atomic jump rates for isolated point defects, but only under very special symmetry conditions as given by Rules 1 and 2, or by the elastic dipole method, which is believed to give the same results so far as indicating the possible presence or absence of mechanical relaxation.

## Footnotes

1 Figures in brackets indicate the literature references at the end of this paper.
